# Can hematological parameters in type 2 diabetes predict microvascular complication development?

**DOI:** 10.12669/pjms.35.6.1150

**Published:** 2019

**Authors:** Erhan Onalan, Nevzat Gozel, Emir Donder

**Affiliations:** 1 Erhan Onalan, Department of Internal Medicine, Faculty of Medicine, Firat University, 23000, Elazig, Turkey; 2 Nevzat Gozel, Department of Internal Medicine, Faculty of Medicine, Firat University, 23000, Elazig, Turkey; 3 Emir Donder, Department of Internal Medicine, Faculty of Medicine, Firat University, 23000, Elazig, Turkey

**Keywords:** Diabetes mellitus, Neutrophil/lymphocyte ratio, Platelet/lymphocyte ratio, Haemoglobin A1c

## Abstract

**Objective:**

To examine potential associations between neutrophil/lymphocyte ratio, platelet/lymphocyte ratio, mean platelet volume (MPV), HbA1c and microvascular complications in diabetic patients from a cost-effectiveness perspective.

**Methods:**

One hundred patients with type 2 diabetes attending our outpatient unit between May 2018 and October 2018 were included, and 100 healthy individuals served as the control group. A retrospective file search was performed to collect information on hemoglobin, mean platelet volume (MPV), glycosylated haemoglobin (HbA1c), hematocrit (Hct), neutrophil and lymphocyte count, neutrophil/lymphocyte ratio (NLR), platelets (Plt), platelet/lymphocyte ratio (PLR), and microvascular complications (neuropathy, retinopathy, nephropathy).

**Results:**

Demographic and laboratory data were retrospectively controlled between diabetes (n=100) and healthy control (n=100) groups. The mean age in diabetic patients and healthy controls was 56.34 and 36.68 years, respectively. The mean NLR in diabetics and healthy controls was 2.48 and 2.11, the difference in NLR being significant (p=0.002). MPV in diabetics and controls was 8.54 and 8.53, respectively, and the difference was not significant (p=0.93). PLR was also similar, i.e. 149.7 and 145.3 in diabetics and healthy controls (p=0.067). With respect to microvascular complications, retinopathy was found to be significantly associated with MPV and NLR (p=0.015, and p=0.051), and nephropathy showed a significant association with NLR (p=0.027) among diabetics. In contrast with the two other microvascular complications, no significant association between neuropathy and NLR could be detected, while PLR and neuropathy was significantly associated (p=0.003).

**Conclusion:**

Microvascular complications may be associated with certain hematologic parameters, as suggested by comparisons both between diabetics and healthy individuals and within the group of diabetic individuals. We believe that hematologic parameters such as hematocrit, MPV, NLR, and PLR, which can be obtained through a simple complete blood count, may be utilized as cost-effective predictors of diabetic microvascular complications. Further prospective studies with larger sample size are required to better delineate these associations.

## INTRODUCTION

Diabetes mellitus (DM) is a systemic and chronic metabolic disorder leading to chronic hyperglycemia that is characterized by disturbances by carbohydrate, protein and fat metabolism due to partial or complete lack of insulin and/or insulin resistance. Chronic hyperglycemia may not only lead to acute metabolic complications, but also long-term injury and dysfunction in several organs and systems of the body, particularly the eyes, kidneys, heart, and blood vessels.^1^

Neutrophil/lymphocyte ratio (NLR), platelet/lymphocyte ratio (PLR), and platelet indices are inexpensive, practical, and easily accessible parameters readily estimated from complete blood count that have been found to be related with a number of medical conditions and pathologies.^2^-^4^ Studies reported correlations between these ratios and indices and metabolic/endocrinologic disorders.^5^,^6^ Inflammatory processes play a key role in chronic diseases in many chronic conditions including cardiovascular disease, cancer, chronic kidney disease, and diabetes mellitus.^7^ Previous studies showed that the neutrophil/lymphocyte ratio (NLR) represents a systemic marker of inflammation, with a significant role in predicting the short- and long-term mortality for cardiovascular conditions and in predicting the prognosis in cancer patients.^8^,^9^

This study was undertaken to assess the utility of these simple, widely available, and inexpensive CBC parameters as markers of microvascular complications of diabetes and as predictors of potential complications.

## METHODS

The study protocol was approved by the Ethics Committee approval of the Scientific Research Coordination Unit on 22.11.2018, with the reference number 08 at Fırat University. Data were retrospectively collected for patients attending to the outpatient unit of General Internal Medicine, Medical Faculty of Fırat University between May 2018 and October 2018. In addition to 100 study subjects with diabetes, 100 healthy individuals served as controls. Patients were included if they had no diagnosed conditions other than diabetes, were between 30-75 years of age, and were receiving oral anti-diabetics. Exclusion criteria were the presence of concomitant conditions (e.g. coronary artery disease, hematological conditions, malignancy, severe hepatic disorder, severe renal disorder), insulin use, and current smoking. A retrospective patient file search was performed to retrieve information on hemoglobin, mean platelet volume (MPV), glycosylated hemoglobin (HbA1c), hematocrit (Hct), neutrophil and lymphocyte count, neutrophil/lymphocyte ratio (NLR), platelets (Plt), platelet/lymphocyte ratio (PLR), and microvascular complications (neuropathy, retinopathy, nephropathy). Diabetic patients with a + proteinuria and/or a creatinine level > 1.2 mg/dl were deemed to have diabetic nephropathy, and those diagnosed with diabetic retinopathy at the Department of Ophthalmology were deemed to have diabetic retinopathy. Patients with findings of polyneuropathy or neuropathy in EMG were considered to have diabetic neuropathy. The subset of patients with microvascular complicationts consisted of those with nephropathy, retinopathy, and/or neuropathy. Demographic data for the healthy controls and patients (i.e. age, gender, waist and hip circumference, body mass index) were collected from patient files.

### Statistical Analyses

All statistical analyses were performed using Statistical Package for the Social Sciences (SPSS) 22.0 software pack. For data assessment, in addition to descriptive statistics [Mean (*X̄*), Standard deviation (SD)], Student’s t test was used for the comparison of quantitative data with normal distribution, while one-way ANOVA was used for the comparisons between the groups. The significance of the difference between two statistically matched samples was tested with Wilcoxon’s paired two sample test, while the chi-square test was used for the comparison of quantitative data. The results were expressed with 95% confidence intervals, and a p value of less than 0.05 was considered significant.

## RESULTS

The comparison between diabetics and healthy controls with respect to demographic and laboratory parameters is shown in Table-I. The mean age in the diabetic group and healthy controls was 54.34 and 36.68 years, respectively. The mean NLR in diabetics and healthy controls was 2.48 and 2.11, the difference in NLR being significant (p=0.002). MPV in diabetics and controls was 8.54 and 8.53, respectively, and the difference was not significant (p=0.93). PLR was also similar, i.e. 149.7 and 145.3 in diabetics and healthy controls (p=0.067).

**Table I:** Demographic and laboratory data in diabetic patients and healthy controls.

	Groups	N	Mean	Standard Deviation	p value
Gender (M/F)	healthy	46/54			
	diabetic	48/52			
Age (year)	healthy	100	36.68	10.756	<0.001
	diabetic	100	56.34	12.554	
HbA1c (%)	healthy	100	5.718	0.2938	<0.001
	diabetic	100	9.624	2.2356	
Hematocrit (%)	healthy	100	41.676	5.6697	0.736
	diabetic	100	41.376	6.8421	
Platelet (x10^9^/L)	healthy	100	261620	55441.985	0.052
	diabetic	100	279390	71906.771	
Neutrophil (x10^9^/L)	healthy	100	4081.00	1455.345	0.012
	diabetic	100	4684.80	1888.585	
Lymphocyte (x10^9^/L)	healthy	100	1972.40	452.028	0.044
	diabetic	100	2201.70	1034.883	
MPV (fl)	healthy	100	8.530	0.9422	0.930
	diabetic	100	8.541	0.8186	
NLR (%)	healthy	100	2.1176	0.81013	0.022
	diabetic	100	2.4823	1.35413	
PLR (%)	healthy	100	145.392	58.5490	0.677
	diabetic	100	149.724	85.8579	

NLR: Neutrophil/lymphocyte ratio; PLR: Platelet/lymphocyte ratio; MPV: Mean platelet volume.

With respect to microvascular complications, retinopathy was found to be significantly associated with MPV and NLR (p=0.015, and p=0.051), and nephropathy showed a significant association with NLR (p=0.027) among diabetics. In contrast with the two other microvascular complications, no significant association between neuropathy and NLR could be detected, while PLR and neuropathy was significantly associated (p=0.003) (Table-II).

**Table II:** The association of diabetic microvascular complications with MPV, NLR, and PLR.

	Retinopathy	N	Mean	Standard Deviation	P value
MPV (fl)	Present	28	8,22	0,8562	0,015
	Absent	72	8.66	0.7752	
NLR (%)	Present	28	2.90	1.5572	0.051
	Absent	72	2.31	1.2397	
PLR (%)	Present	28	146.8	64.254	0.834
	Absent	72	150.8	93.295	

	*Nephropathy*				

MPV (fl)	Present	22	8.30	0.791	0.118
	Absent	78	8.60	0.818	
NLR (%)	Present	22	3.04	1.477	0.027
	Absent	78	2.32	1.280	
PLR (%)	Present	22	149.3	55.390	0.982
	Absent	78	149.8	75.374	

	*Neuropathy*				

MPV (fl)	Present	74	8.47	0.784	0.198
	Absent	26	8.71	0.902	
NLR (%)	Present	74	2.39	1.293	0.280
	Absent	26	2.73	1.019	
PLR (%)	Present	74	134.7	56.90	0.003
	Absent	26	192.4	131.05	

NLR: Neutrophil/lymphocyte ratio; PLR: Platelet/lymphocyte ratio; MPV: Mean platelet volume.

## DISCUSSION

Neutrophil/lymphocyte ratio (NLR), platelet/lymphocyte ratio (PLR), and the platelet index represent inexpensive, practical, and widely available laboratory parameters that can be readily estimated from routine complete blood counts. The association between these parameters and many medical conditions and pathologies has been clearly established.^2^-^4^ Also, these indices and ratios have been reported to be associated with several metabolic and endocrinologic disorders.^5^,^6^

In our study, there was a significant difference in terms of NLR between diabetics and healthy controls (p=0.002).In many epidemiological studies, chronic inflammation has been reported to play a significant role in the pathogenesis of chronic diseases such as metabolic syndrome, hypertension, and diabetes.^10^,^11^ Cross-sectional and prospective studies also have indicated a positive correlation between Type 2 diabetes and its complications and C-reaktif protein(CRP), Interleukin-6(IL-6), and leukocyte counts.^12^

One study examining the association between gestational diabetes and mean platelet volume (MPV) showed significantly higher MPV values women with gestational diabetes as compared to those without this condition. Furthermore, MPV was reported to be correlated with the insulin resistance index, i.e. (Homeostatic Model Assessment for Insulin Resistance) HOMA-IR.^13^ While significant correlations between neuropathy and MPV were observed in some studies, in the study by Hekimsoy et al. involving 145 diabetic and 100 non-diabetic individuals, no statistically significant differences were foun.^14^,^15^ Similarly, we failed to detect differences in MPV between diabetics and healthy controls in our study. On the other hand, increased NLR values were found to represent a weak prognostic indicator in patients undergoing cardiovascular interventions. In some studies, elevated NLR was also found to be linked with increased mortality.^14^,^15^ For instance, a study examining the effect of cigarette smoking, several indices including NLR, PLR and the platelet index were higher among smokers, and NLR and platelet values were reported to be associated with the intensity of smoking.^15^ Therefore, such factors should be taken into consideration when assessing NLR, PLR, and other similar indices.

In the current study, a similar connection between NLR and the microvascular complications of diabetes such as retinopathy and nephropathy was observed, while no such correlations could be identified for neuropathy. Again, MPV and NLR were significantly associated with retinopathy and NLR with nephropathy. In contrast with the two other microvascular complications, neuropathy did not significantly correlate with NLR, while PLR and nephropathy were significantly associated. The similarities and disagreements between our results and previous publications may be partially explained by the limitations of our study. Also, absence of a homogeneous distribution of the patients with regard to age, gender, oral antidiabetic use, and body mass index in this cross-sectional study represents one such limitation.

### Limitations of the study

Our study should be evaluated in the light of several limitations. The presented study was conducted on a retrospective basis and represented single-center experience.

## CONCLUSION

In conclusion, based on the comparisons between diabetic patients and healthy controls and within the group of diabetic individuals, our results seem to suggest that microvascular complications may be linked with certain hematological parameters in type 2 DM patients. Hematocrit, MPV, NLR, and PLR values have been found to be cost-effective parameters that predict diabetic microvascular complications and that can be readily estimated using a simple laboratory test such as the complete blood count. Further studies with larger sample size are warranted to better elucidate the agreement and inconsistency between our results and previous publications, and to reach firmer conclusions regarding the value of these indices.

### Authors Contribution:

**EO:** Conceived, designed, did statistical analysis and manuscript writing.

**EO & NG:** Did data collection and manuscript review.

**ED:** Did manuscript review and final approval.
